# Mechanisms of Tumor Necrosis Factor-Alpha Inhibitor-Induced Systemic Lupus Erythematosus

**DOI:** 10.3389/fmed.2022.870724

**Published:** 2022-06-06

**Authors:** Chung-Yang Yen, Sheng-Jie Yu, Yi-Ming Chen, Kuo-Lung Lai, Yi-Da Wu, En-Chih Liao, Ching-Liang Hsieh

**Affiliations:** ^1^Department of Dermatology, Taichung Veterans General Hospital, Taichung City, Taiwan; ^2^School of Medicine, National Yang Ming Chiao Tung University, Taipei, Taiwan; ^3^Department of Education and Research, Kaohsiung Veterans General Hospital, Kaohsiung City, Taiwan; ^4^Division of Allergy, Immunology and Rheumatology, Department of Internal Medicine, Taichung Veterans General Hospital, Taichung City, Taiwan; ^5^Department of Medicine, MacKay Medical College, New Taipei City, Taiwan; ^6^Chinese Medicine Research Center, China Medical University, Taichung City, Taiwan; ^7^Department of Chinese Medicine, China Medical University Hospital, Taichung City, Taiwan

**Keywords:** systemic lupus erythematosus, tumor necrosis factor-alpha inhibitors, IL-2, IL-10, IFN-γ/IL-13 ratio

## Abstract

Systemic lupus erythematosus induced by biologics mainly results from tumor necrosis factor-alpha remains unclear. The objectives of the study were to investigate the mechanisms of tumor necrosis factor-alpha inhibitor-induced systemic lupus erythematosus. Peripheral blood mononuclear cells obtained from thirteen psoriasis patients were cultured and treated with the following: untreated control, *Streptococcus pyogenes* with or without different biologics. The supernatants were collected for cytokines assay. Analysis of cytokine expression revealed that IL-2 and IL-10 levels decreased only in the TNF-α inhibitor-treated groups but not in the groups treated with biologics involving IL-17, IL-12/IL-23 or IL-23 inhibitor mechanisms (*p* < 0.001, *p* < 0.05). The IFN-γ/IL-13 ratio increased significantly in patients with SLE inducing biologics to *S. pyogenes* induction only compared with non-SLE inducing biologics to *S. pyogenes* induction only (*p* = 0.001). IL-2 and IL-10 depletion and a shift to the Th-1 pathway in the innate response are the correlated mechanism for tumor necrosis factor-alpha inhibitor-induced systemic lupus erythematosus.

## Introduction

Biologics are widely used in treating moderate to severe psoriasis, psoriatic arthritis, rheumatoid arthritis, and hidradenitis suppurativa (1–4). Systemic lupus erythematosus (SLE) induced by biologics mostly occurs during the treatment of psoriasis with tumor necrosis factor-alpha (TNF-α) inhibitors, especially in patients with psoriatic and rheumatoid arthritis. TNF-α inhibitors were calculated with an odds ratio of 3.64 for SLE development (5). The onset of symptoms ranges from <1 month to more than 4 years. High antinuclear antibody (ANA) levels, double stranded DNA (dsDNA) autoantibody positivity, thrombocytopenia, leukopenia, hypocomplementemia, skin rash, and arthritis are relatively common symptoms in TNF-α inhibitor-induced SLE (TAILS) (6). Further pathological findings characterizing classical drug-induced lupus include an increased frequency of significant anti-dsDNA antibody titers and a decreased incidence of anti-histone antibodies (7). The incidence of TAILS in patients with ulcerative colitis/Crohn's, etc., is 0.5–1% (8). However, how anti-TNF-α agents induce autoantibody expression and lead to the development of SLE remain incompletely understood. *Streptococcus pyogenes* (*S. pyogenes*) can trigger the immune responses to activate psoriasis outbreaks (9, 10). Furthermore, the innate immune system has been shown to be activated by *S. pyogenes* in both guttate and chronic plaque psoriasis (11). The specific IgA response against to *S*. *pyogenes* was correlated with a cutaneous lymphocyte-associated antigen^+^ T-cell-depend IL-17F response (12). In this study, peripheral blood mononuclear cells (PBMCs) from psoriasis patients and *S. pyogenes* was used to challenge the PBMCs to simulate a real clinical psoriasis outbreak. We describe the cases of three patients with psoriasis who developed TAILS and investigated the underlying mechanisms. Two of these patients developed TAILS after treatment with adalimumab and one after treatment with etanercept. All three patients met the 1997 American College of Rheumatology (ACR) criteria for SLE.

## Materials and Methods

### Participants

Thirteen psoriasis patients with or without psoriatic arthritis and three healthy controls were enrolled in this study. All participants were selected from the clinic of the Dermatology or Rheumatology Departments of Taichung Veterans General Hospital. All participants provided written informed consent. The protocols and all research involving human participants were approved by the Institutional Review Board of Taichung Veterans General Hospital (TCVGH-CE16265B; TCVGH-CE20043B).

### Materials

*Streptococcus pyogenes* group A was identified and provided by the Department of Pathology and Laboratory Medicine of Taichung Veterans General Hospital. After heat inactivation, *S. pyogenes* group A was placed on blood agar plates for 1 week.

### Cell Culture

For PBMC culture, 16 mL of blood was collected from each patient in sodium citrate tubes (Vacutainer® CPT™, BD, USA), and PBMCs were purified through centrifugation over a density gradient. The cells were washed with PBS and subsequently cultured in RPMI-1640 supplemented with 10% fetal bovine serum and 1% penicillin/streptomycin at 37°C and 5% CO_2_. *S. pyogenes* group A was prepared at a concentration of 2 × 10^7^ CFU/mL under the similar cell viability to normal control and proper immune induction response on PBMC (data not shown).

A total of 6 × 10^5^ cells per milliliter were then cultured in a 12-well plate and treated for 24 h with the following: control, *S. pyogenes* only, *S. pyogenes* + golimumab (0.5 μg per milliliter), *S. pyogenes* + ixekizumab (3.5 μg per milliliter), *S. pyogenes* + ustekinumab (0.25 μg per milliliter), *S. pyogenes* + adalimumab (4 μg per milliliter), *S. pyogenes* + secukinumab (16.7 or 34 μg per milliliter), *S. pyogenes* + guselkumab (1.2 μg per milliliter), and *S. pyogenes* + etanercept (1.9 μg per milliliter). Supernatants were collected for the subsequent measurement of cytokine levels. The concentrations of the biological agents we tested are the trough serum concentrations at a steady-state indicated in the pharmacokinetic section of the reference list.

### Cell Viability Test

The separated peripheral blood mononuclear cells **(**PBMCs) were cultured using different concentrations of *S. pyogenes* for 24 h, and then, 0.5 mg per milliliter of 3-(4,5-dimethylthiazol-2-yl)-2,5-diphenyltetrazolium bromide was added. After reacting for 1 h, the mixtures were centrifuged and the supernatants were removed. Then, 200 μL of dimethyl sulfoxide was added to lyse the cells and dissolve purple crystals, and cell viability was analyzed using an enzyme-linked immunosorbent assay reader at a wavelength of 570 nm.

### Multiplex Assay for Cytokine Levels

To measuring cytokine levels, culture supernatants were collected, and the concentrations of IL-2, IL-4, IL-5, IL-6, IL-7, IL-8, IL-9, IL-10, IL-12, IL-13, IL-17A, IFN-γ, and TNF-α were determined using a protein multiplex immunoassay system (Bio-Plex Cytokine Array System, Bio-Rad Laboratories, Hercules, CA, USA). The IFN-γ/IL-13 ratio was calculated in different patients undergoing different biologics *in vitro*. If the patient uses this biological agent and eventually causes SLE, it can be classified into the SLE group. Conversely, if the patient uses another biological agent lead to eventually doesn't get SLE, it can be classified as a non-SLE group.

### Statistical Analyses

All statistical analyses were performed using SPSS version 22 (IBM, Armonk, NY, U.S.A.). Analysis of cytokine expression was with the use of the Mann-Whitney U test. The ratio of IFN-γ to IL-13 between TAILS-inducing biologics and non-TAILS-inducing biologics was analyzed with *t*-test. Data were presented as the mean ± standard deviation (SD). Two-sided *P*-values of 0.05 or less were considered to indicate statistical significance.

## Results

### Patients

Thirteen patients were enrolled in our study including, three of which developed TAILS that met the 1997 ACR criteria for SLE. Patients 1 and 2 received adalimumab for 6 and 47 months, respectively, whereas Patient 3 received etanercept for 24 months (Table 1). In addition, we assessed the patients' skin condition by using the Psoriasis Area and Severity Index (PASI). Patient 1's psoriasis worsened after adalimumab treatment from absolute PASI:15 to PASI:16; Patient 2's psoriasis was largely stable, going from PASI:3.8 to PASI:2; and Patient 3's psoriasis improved, going from PASI:26 to PASI:13 (Table 2). Of the three cases, Patient 1 experienced an outbreak of psoriasis and progressed to SLE most quickly. TNF-α inhibitor administration was subsequently discontinued for all three patients.

**Table 1:** Demographic and clinical characteristics of psoriasis patients (*n* = 13).

**Pt**	**Age/gender**	**PSO**	**PSA**	**Time of PBMCs test**	**Course of TNF-α inhibitor**	**ANA dsDNA**	**HBV HCV**	**Other systemic disease**	**TAILS**
P1	42y/ M	+	+	2 months after TAILS	6 months of adalimumab	+ +	– –	Alcoholic hepatitis	+
P2	56y/ F	+	+	1 month after TAILS	47 months of adalimumab	+ +	– –	–	+
P3	69y /M	+	+	16 months after TAILS	24 months of etanercept	+ +	– –	–	+
P4	50y/ M	+	+	6th month of guselkumab	26 months of adalimumab	– –	– –	–	–
P5	38y/ M	+	–	3th month of ixekizumab	No	– –	– –	–	–
P6	58y/ M	+	+	12th month of adalimumab	12 months of adalimumab	+ –	– –	–	–
P7	64y/ F	+	+	15th month of secukinumab	52 months of adalimumab	– –	– –	–	–
P8	54y/ F	+	+	29th month of golimumab	30 months of golimumab 12 months of adalimumab	– –	– –	–	–
P9	44y/ M	+	–	5th month of adalimumab	20 months of adalimumab	+ –	– –	–	–
P10	53y/ M	+	–	1st month of ixekizumab	No	– –	+ –	–	–
P11	67y/ F	+	–	21th month of secukinumab	23 months of adalimumab	– –	– –	–	–
P12	39y/ M	+	–	19th month of ustekinumab	No	+ –	– –	–	–
P13	53y/ F	+	–	10th month of ixekizumab	21 months of adalimumab	– –	– –	–	–

*Pt, Patient; PSO, Psoriasis; PSA, Psoriatic arthritis; HBV, Hepatitis B virus; HCV, Hepatitis C virus; TAILS, TNF-α inhibitor-induced systemic lupus erythematosus*.

**Table 2:** Clinical characteristics of patients with TNF-α inhibitor-induced systemic lupus erythematosus.

**Patients**	**TAILS autoantibodies**	**Thrombocytopenia**	**C3, C4**	**Lymphopenia**	**PASI**
P1	ANA, ACA Anti-dsDNA, Anti-RNP	+	Low	–	15 → 16
P2	ANA, Anti-LA Anti-dsDNA	–	–	+	3.8 → 2
P3	ANA, Anti-SSA Anti-dsDNA	+	Low	–	26 → 13

*Anti-dsDNA, anti-double stranded DNA; Anti-LA, anti-lupus anticoagulant; Anti-SSA, anti-Sjögren's syndrome-related antigen A; ACA, anti-cardiolipin antibodies; Anti-RNP, anti-nuclear ribonucleoprotein; C3, complete 3; C4, complete 4; PASI, Psoriasis Area and Severity Index; ANA, antinuclear antibody*.

### Response to Treatment

Patient 1 refused oral prednisolone and received ustekinumab for psoriasis and subsequently recovered in skin condition, going from PASI:16 to PASI:0.2. Patient 2 received secukinumab (150 mg per week), following which skin absolute PASI declined to 0. Patient 3 received Methotrexate (7.5 mg per week) for controlling psoriasis and thrombocytopenia, and skin absolute PASI was maintained to 18.2. ANA remained positive in all three cases. In addition, dsDNA autoantibody titer levels remained high in Patient 1 after 6 months of ustekinumab treatment. The level of the dsDNA autoantibody titer declined to normal in Patient 2 after only 2 months and after 1.5 years in Patient 3.

### Cytokine Expression Analysis

**Figure 1:**
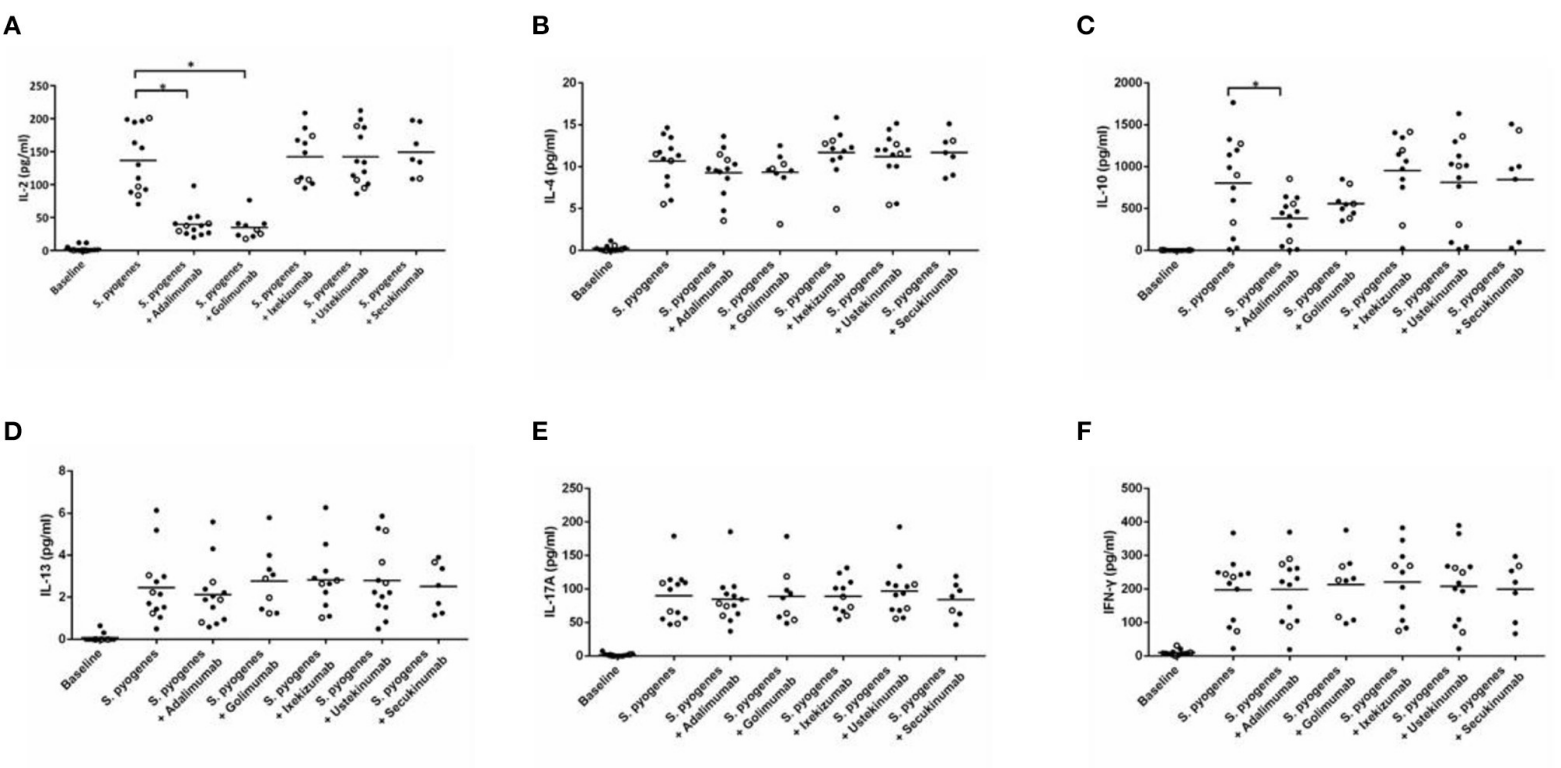
Cytokine expression in peripheral blood mononuclear cells after induction with *S. pyogenes* and treated with different biologics. **(A–F)** depict the levels of cytokines (shown on the y-axis) in response to the application of listed biologics (shown on the x-axis) after induction by *S. pyogenes*. Each circle represents cell cultures from one patient. In all panels, the three TNF-α inhibitor-induced SLE patients are marked with hollow circles. Black bars in each column indicate the mean cytokine concentration. TNF-α inhibitors significantly inhibited *S. pyogenes*-induced IL-2 **(A)** (*p* < 0.001 in adalimumab, *p* < 0.001 in golimumab, respectively) and IL-10 **(C)** expression (*p* = 0.039 in adalimumab).

**Figure 2:**
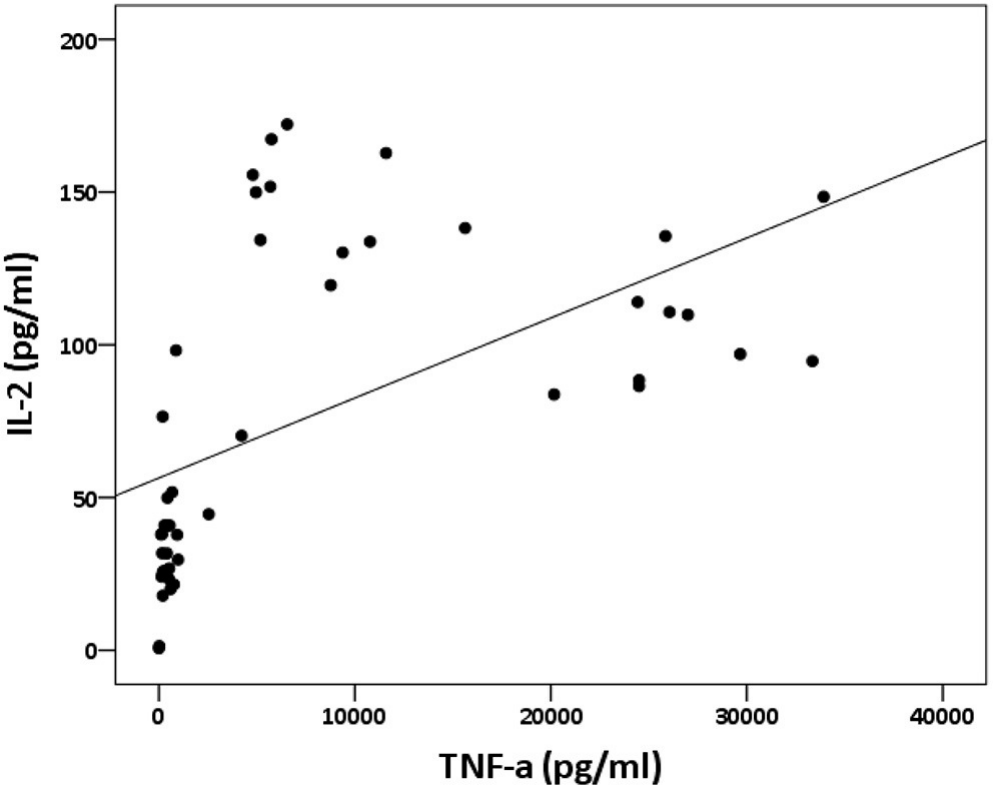
Relation between IL-2 and TNF-α concentration *in vitro*. The panel depicts the levels of IL-2 (shown on the y-axis) and the levels of TNF-α (shown on the x-axis). The data between the concentration of IL-2 and TNF-α *in vitro* is a proportional correlation (*p* < 0.000; rs = 0.74).

**Figure 3:**
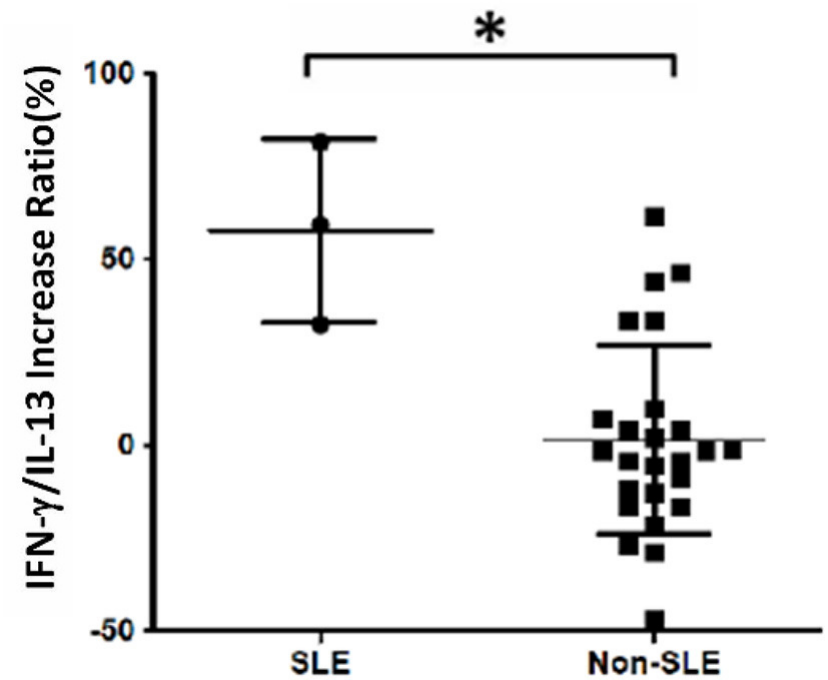
Increased IFN-γ/IL-13 ratio in patients with systemic lupus erythematosus (SLE). Cell samples were obtained from 13 patients, and 28 treatment courses of biologics were as follows: adalimumab, 9; etanercept, 1; golimumab, 1; ustekinumab, 8; ixekizumab, 3; secukinumab, 5; and guselkumab, 1. The increase in the ratio of IFN-γ to IL-13 levels was calculated as the increase in percentage following treatment with biologics compared with *S. pyogenes* induction only: (IFNγ/IL13 w/S. pyogenes & biologics) − (IFNγ/IL13 w/S. pyogenes only)/(IFNγ/IL13 w/S. pyogenes only). We found that the ratio was significantly increased in the SLE group (*p* = 0.001).

The analysis of cytokine levels on psoriasis patients and healthy controls revealed no obvious difference (Supplementary Table S1). The analysis of cytokine expression treated with experienced or naïve biological agents revealed that IL-2 and IL-10 levels decreased in only the TNF-α inhibitor-treated groups but not in the groups treated with biologics involving inhibitor mechanisms for IL-17, IL-12/IL-23 or IL-23 (*p* < 0.001, *p* < 0.05, respectively; Figure 1). IL-2 and IL-10 levels also decreased in only the TNF-α inhibitor-treated groups but not in the groups treated with other biologics in healthy controls (Supplementary Table S1). The data between the concentration of IL-2 and TNF-α *in vitro* is a proportional correlation (Figure 2). We hypothesize that IL-2 secreting requires not only pathogens but also TNF-α, explaining IL-2 depletion in the presence of TNF-α inhibitors. The hypothesis needs more evidence to prove. The anti-IL-17 inhibitors, including ixekizumab and secukinumab, apparently did not attenuate IL-17, which could result from the action of different epitopes used for verifying the concentration in the multiplex assay and their blocking by biologics. In our thirteen patients, most of them experienced two or more biologics. Twenty-eight clinical treatment courses of biologics in total were as follows: adalimumab, 9; etanercept, 1; golimumab, 1; ustekinumab, 8; ixekizumab, 3; secukinumab, 5; and guselkumab, 1. The ratio of IFN-γ to IL-13 was significantly higher (*p* = 0.001) between after induction with *S. pyogenes* plus SLE-inducing biologics and after induction with *S. pyogenes* only than after induction with *S. pyogenes* plus non-SLE-inducing biologics and after induction with *S. pyogenes* only (Table 3; Figure 3). Because three patients with SLE were PSO + PSA, only four patients with PSO+PSA without SLE (P4, P6, P7, P8) were selected and the ratio of IFN-γ/IL13 was also significantly increased in the SLE group (*p* = 0.02; Supplementary Figure S1). This ratio could be used in the future for identifying the risk of TAILS. Taken together, low levels of TNF-α was correlated to low levels of IL-2. IL-2 and IL-10 depletion could create an environment for developing SLE. The ratio of IFN-γ/IL13 in SLE-inducing biologics was significantly higher than in non-SLE-inducing biologics.

**Table 3:** Laboratory profile of psoriasis patients with administered biologics (*n* = 13).

**Pt**	***S. pyogenes* induction and treated with experienced biologics**	**IL-2** **(pg/ml)**	**IL-4 (pg/ml)**	**IL-10** **(pg/ml)**	**IL-17A** **(pg/ml)**	**TNF-a** **(pg/ml)**	**IFN-r (pg/ml)**	**IL-13** **(pg/ml)**	**IFN-r/ IL13**
P1	Baseline	OOR <	OOR <	4.7	0.52	8.77	1.1	OOR <	
	*S. pyogenes*	200.97	5.54	334.27	109.09	OOR>	74.46	1.24	60.05
	Adalimumab	40.85	3.56	115.98	78.53	544.03	88.28	0.81	108.99
	Ustekinumab	188.94	5.45	309.58	107.2	OOR>	71.02	1.24	57.27
P2	Baseline	OOR <	0.14	4.15	1.51	9.93	10.71	OOR <	
	*S. pyogenes*	83.76	10.73	900.4	48.43	20,165.1	244.94	3.05	80.3
	Adalimumab	29.67	10.82	556.83	60.28	978.96	290.14	2.73	106.28
	Secukinumab150	109.2	13.1	1,435.6	68.12	OOR>	269.05	4.27	63.01
P3	Baseline	0.69	0.59	7.5	2.52	25.73	31.3	OOR <	
	*S. pyogenes*	96.95	11.5	1,275.85	66.79	29,662.5	236.09	2.24	105.4
	Etanercept	44.56	9.63	670.77	53.09	2,542.91	241.47	1.44	167.68
P4	Baseline	0.52	0.33	3.32	1.85	15.73	8.94	OOR <	
	*S. pyogenes*	92.53	12.17	749.77	56.56	OOR>	217.19	1.53	141.95
	Adalimumab	20.03	9.41	297.32	53.09	591.14	232.06	1.53	151.67
	Guselkumab	100.48	12.34	699.96	58.84	OOR>	208.68	1.34	155.73
	Secukinumab300	108.58	12.94	852.81	63.29	OOR>	220.42	1.71	128.9
P5	Baseline	0.86	0.05	2	1.18	13.5	9.37	OOR <	
	*S. pyogenes*	88.45	11.74	1,142.72	65.1	24,502.4	273.76	2.15	127.33
	Ixekizumab	94.67	12.17	1,067.13	71.14	33,347.4	297.09	2.24	132.63
	Ustekinumab	86.45	12.02	1,016.31	69.45	24,502.4	268.46	2.24	119.85
P6	Baseline	11.93	0.51	3.14	7.68	36.39	9.93	0.65	
	*S. pyogenes*	194.6	13.94	1,768.01	179.24	OOR>	200.39	5.19	38.6
	Adalimumab	98.18	13.64	643.06	185.8	874.18	222.11	4.31	51.53
	Ustekinumab*	212.43	14.48	1,636.57	193.12	OOR>	201.66	5.28	38.19
P7	Baseline	1.17	OOR <	3.94	OOR <	9.84	3.67	OOR <	
	*S. pyogenes*	198.79	11.35	1,199	107.22	OOR>	85.68	2.74	31.27
	Ustekinumab	198.92	11.43	1,128.8	108.74	OOR>	89.51	2.91	30.76
	Adalimumab	51.75	9.76	631.24	92.9	676.92	102.91	2.04	50.45
	Secukinumab300	197.7	11.23	1,004.17	105.91	OOR>	66.92	2.57	26.04
P8	Baseline	11.79	0.24	2.61	2.03	30.16	6.65	OOR <	
	*S. pyogenes*	196.47	10.94	991.2	99.62	OOR>	107.72	2.99	33.69
	Golimumab	38.01	8.72	587.27	87.07	163.46	107.68	2.22	48.5
	Adalimumab	49.92	8.62	465.08	84.69	437.61	105.21	2.22	47.39
P9	Baseline	OOR <	OOR <	0.88	OOR <	3.98	1.87	OOR <	
	*S. pyogenes*	130.23	6.01	11.76	109.76	9,378.42	22.9	0.5	45.8
	Adalimumab	37.91	4.76	12.98	103.76	110.07	19.71	0.59	33.4
	Ustekinumab	119.51	5.6	13.8	103.76	8,766.19	21.91	0.5	43.82
P10	Baseline	5.22	0.09	2.49	2.52	14.06	7.54	0.32	
	*S. pyogenes*	109.83	13.51	1,328.04	114.16	26,993.3	367.78	6.14	59.9
	Ixekizumab	110.73	13.81	1,348.89	101.38	26,057.7	383.17	6.27	61.1
	Ustekinumab	113.99	13.31	1,300.42	105.09	24,429.7	365.71	5.86	62.4
P11	Baseline	OOR <	OOR <	OOR <	OOR <	3.31	6.41	OOR <	
	*S. pyogenes*	70.23	7.77	599.07	47.3	4,220.9	247.16	1.71	144.54
	Adalimumab	31.71	9.77	449.63	89.98	405.98	262.11	2.06	127.24
	Secukinumab300	162	15.15	1,510.21	119.34	OOR>	297.59	3.9	76.31
P12	Baseline	1.36	1.16	1.06	3.58	17.57	21.22	OOR <	
	*S. pyogenes*	163.48	14.67	138.76	113.98	OOR>	249.87	1.44	173.52
	Ustekinumab	135.58	12.02	69.49	93.55	25,847	194.12	0.84	231.1
	Secukinumab300	138.23	11.7	99.67	89.37	15,625.6	188.89	1.25	151.11
P13	Baseline	0.69	OOR <	0.49	OOR <	2.08	12.15	OOR <	
	*S. pyogenes*	155.65	8.82	28.71	55.42	4,794.36	243.3	1.05	231.71
	Adalimumab	24.09	6.85	4.61	37.11	139.35	146.6	0.74	198.11
	Ixekizumab	167.3	9.68	23.49	54.46	5,744.19	250.81	1.1	228.01
	Ustekinumab	172.19	10.1	43.17	68.18	6,545.58	267	1.62	164.81

*Pt, Patient; OOR <, Out of Range Below; OOR>, Out of Range Above*.

*Patient 2 received secukinumab 150 mg monthly and patient 4, 7 received secukinumab 300 mg monthly. The patients' PBMCs were treated with secukinumab 16.7 and 34 μg per milliliter, respectively*.

Mark * means biologics course after PBMCs test.

## Discussion

Decreased expression of IL-2 and IL-10 is associated with SLE development. A lack of IL-2 leads to the inhibition of activation-induced cell death and enhances the longevity of autoreactive T cells (13). Vaccinia recombinant viruses expressing the human IL-2 gene showed prolonged survival, decreased autoantibody in SLE mice (14). Limited IL-10 expression can increase the secretion of proinflammatory cytokines in pathogenic T cells and upregulate the presentation of antigens by dendritic cells to naïve T cells, thus promoting differentiation into pathogenic cells (15). Yin et al. (16) discussed the protective role of IL-10 in the development of lupus. They found that IL-10 depletion was closely related to severe and early-onset lupus and was also associated with IFN-γ production and an increased serum concentration of anti-dsDNA autoantibodies, similar to our observations in patients with TAILS.

As a chronic autoimmune disease, SLE is characterized by the presence of nuclear antigen autoantibodies. Recent studies have implicated innate immunity as a key switch for coordinating B cells, T cells, and macrophages in the pathogenesis of SLE (17). Innate immunity and the T_h_1 reaction are major initiation responses against infections including streptococcus, staphylococcus, and various viruses. In a second aspect, cytokines involved in T_h_2 immunity, including IL-4 and IL-13, counteract the T_h_1 response. Macrophage polarization is a key step that drives the immune response toward either the M1 or M2 pathway. In the early stages of lupus, the M1 pathway is mediated by cytokines including IFN-γ, whereas the M2 pathway is mediated by IL-4 and IL-13. IFN-γ induction favors the production of M1 macrophages that are involved in inflammation and tissue damage. An increased concentration of IFN-γ can alter the ratio of T_h_1 to T_h_2 with predominance by T_h_1 cells (18). In lupus immunopathology, IFN-γ is elevated in the serum of lupus patients, particularly those in the active stage (19). Th1/Th2 balance shift and elimination of IFN-gamma by IFN-γ targeting gene therapy was developed in treating SLE (20). However, the role of T_h_2 immune responses in autoimmune development remains controversial, although it is known that IL-4 plays a crucial role in the Treg-mediated suppressive immune response. Plasma IL-4 concentrations were found to be significantly lower in SLE patients than in healthy controls (21). Furthermore, IL-4 can downregulate T_h_1-mediated IgG subclasses of autoantibodies to prevent the development of lupus-like autoimmune disease (22). Although IL-13 is a strong anti-inflammatory cytokine that modulates macrophages, monocytes, and lymphocytes (23), a previous study found that plasma IL-13 levels were significantly higher in SLE patients than in controls (24). We hypothesize that different stages and timing are key factors governing the IL-13 concentration. Moreover, a disproportionate increase in IFN-γ or reduced IL-13 could be the mechanism in SLE. These differences could be due to possible feedback mechanism *in vivo* (24), which needs to be investigated by further or future studies. Alternatively, this could be due to different evolution—primary SLE and drug-induced SLE (TAILS).

In the present study, the IL-13 concentration increased within 24 h following the onset of inflammation caused by streptococcus infection. However, we are uncertain whether IL-13 promotes inflammation, rather than acting as a feedback cytokine that reduces inflammation. In our study, different biologics appeared to react differently to IL-13, but the detailed mechanisms remain unknown. In the three TAILS patients, we observed the lowest IL-13 concentrations for pathogenic drugs compared with other biologics including anti-IL-17, anti-IL-23, and other anti-TNF-α. IL-17 is apparently an important cytokine in the pathogenesis of SLE because it can amplify the immune response by increasing autoantibodies through B-cell stimulation (25). However, our data indicated no obvious differences in the IL-17 concentration between SLE and non-SLE groups. Anti-IL-17 inhibitors, including ixekizumab and secukinumab, apparently did not attenuate IL-17, which could result from the action of different epitopes used for verifying the concentration in the multiplex assay vs. blocking by biologics.

Adaptive transfer to M2 macrophages reduces the severity of SLE, whereas IL-4 and IL-13 drive the production of M2 macrophages (26). A similar condition was observed in innate lymphoid cells (ILCs). Group 2 ILCs produce IL-4, IL-9, and IL-13. Hou et al. found significantly reduced numbers of Group 2 ILCs in SLE patients (27). Restoring Group 2 ILCs with IL-33 reduced immune cell infiltration and improved survival in a mouse model (28). During outbreaks of psoriasis and SLE, the ratio of M1 to M2 macrophages is increased (26, 29). Other recent studies have highlighted M2 promotion and immunomodulation as another avenue for SLE treatment (30, 31). Hence, maintaining balance of M1/M2 and T_h_1/T_h_2 ratios is an ideal way to prevent SLE. Otherwise, IL-13 induction will favor the production of M2 macrophages that are involved in tissue repair.

In the present study, the data revealed that the three TAILS cases had high ratios of IFN-γ to IL-13 compared with those of biologics targeting other mechanisms in the same patients. Taken together with previous findings, our results suggest that the depletion of IL-2 and IL-10, along with the deterioration of the T_h_1/T_h_2 ratio, are potentially a major underlying mechanism for TAILS. To verify this, we analyzed the symptom severity of the three TAILS patients. Of the three cases, Patient 1's symptoms were the most severe, whereas Patient 2's was the least severe. Patients 1 and 3 were revealed to have low levels of IL-4, whereas Patient 2's IL-4 level was normal when compared with other biologics. Less deviation in the ratio of IFN-γ to IL-13 and a normal level of IL-4 could explain why Patient 2's symptoms were the least severe of the three patients. Hence, the degrees of deviation in the IFN-γ/IL-13 ratio and IL-4 impairment could be regarded as indicators of disease severity.

In the present study, we tested two types of anti-TNF-α (golimumab and adalimumab) in all three patients and an additional anti-TNF-α (etanercept) in Patient 3. The IFN-γ/IL-13 ratio in adalimumab and golimumab was 108.99 and 22.59, respectively, in Patient 1. The IFN-γ/IL-13 ratio in adalimumab and golimumab was 106.28 and 92.69, respectively, in Patient 2. The IFN-γ/IL-13 ratio in adalimumab, golimumab, and etanercept were 145.37, 114.5, and 167.68, respectively, in patient 3. These findings support the previous hypothesis that drug-induced SLE can be drug-specific rather than class-specific (6). Therefore, it is crucial to identify which patients will develop drug-induced SLE and what types of drugs will induce it. We believe the findings presented herein will provide a clear understanding for TAILS. We also believe the incidence of TAILS is underestimated.

## Limitation

This study has some limitations. First, the results of the study need to be validated in a larger group to assess the variability and validity of our findings. Second, *in vitro* experiments on different cells line about cytokine expression, Th1/Th2 related cells subpopulation, and how the mechanisms of different biologics are affected by the IFN-γ or IL-13 concentration requires further investigation.

## Conclusions

In conclusion, our results suggest that the underlying expression of TNF-α inhibitor-induced SLE correlates with depletion of IL-2 and IL-10 and T_h_1/T_h_2 related cytokine imbalance. Our findings provide a clearer understanding of SLE and suggest more appropriate treatments such as low-dose courses of IL-2 and IL-10. Although the sample sized analyzed was small, the observations in this study highlight possible mechanisms of etiology. However, more sample size researches are needed in the future.

## Data Availability Statement

The original contributions presented in the study are included in the article/Supplementary Material, further inquiries can be directed to the corresponding author/s.

## Ethics Statement

The studies involving human participants were reviewed and approved by Institutional Review Board of Taichung Veterans General Hospital (TCVGH-CE16265B; TCVGH-CE20043B). The patients/participants provided their written informed consent to participate in this study.

## Author Contributions

C-YY performed the experiments, protocol design, and wrote the manuscript. S-JY and E-CL performed the experiments. Y-MC, K-LL, and Y-DW participated in protocol design and discussion. C-LH participated in protocol design and revised the manuscript. All authors contributed to the article and approved the submitted version.

## Funding

This work was funded in part by the Chinese Medicine Research Center, China Medical University from the Featured Areas Research Center Program within the framework of the Higher Education Sprout Project by the Ministry of Education (MOE) in Taiwan (CMRC-CENTER-0). And also from Ministry of Science and Technology in Taiwan (MOST 109-2320-B-39-044). The funders had no role in study design, data collection and analysis, interpretation of findings, manuscript writing, and target journal selection.

## Conflict of Interest

The authors declare that the research was conducted in the absence of any commercial or financial relationships that could be construed as a potential conflict of interest.

## Publisher's Note

All claims expressed in this article are solely those of the authors and do not necessarily represent those of their affiliated organizations, or those of the publisher, the editors and the reviewers. Any product that may be evaluated in this article, or claim that may be made by its manufacturer, is not guaranteed or endorsed by the publisher.

## Abbreviations

ACR: American College of Rheumatology
ANA: antinuclear antibody
dsDNA: double stranded DNA
IFN-γ: interferon-gamma
IL: interleukin
PASI: Psoriasis Area Severity Index
PBMC: peripheral blood mononuclear cell
SLE: Systemic lupus erythematosus
TAILS: TNF-α inhibitor-induced SLE
Th1: T helper type 1
Th2: T helper type 2
*S. pyogenes*: *Streptococcus pyogenes*.
