# Supplementary material for: Mechanisms of Tumor Necrosis Factor-Alpha Inhibitor-Induced Systemic Lupus Erythematosus
Source: Front Med (Lausanne). 2022 Jun 6;9:870724. doi: 10.3389/fmed.2022.870724 (PMC9208548; doi:10.3389/fmed.2022.870724)
Supplement: Supplementary file 1 [file Data_Sheet_1.pdf]

Appendix A

Table S1. The analysis of cytokine levels on psoriasis patients and healthy controls.

		Total (n=16)			Healthy control (n=3)			Patient (n=13)			*p* value
		n	mean	±SD	n	mean	±SD	n	mean	±SD	
IL-2 (pg/ml)	Baseline	16	3.22	±4.44	3	5.48	±5.13	13	2.69	±4.33	0.138
	S. pyogenes	16	137.57	±50.89	3	139.69	±67.06	13	137.08	±49.86	0.884
	adalimumab	16	40.96	±20.27	3	46.14	±25.12	13	39.77	±20.00	0.587
	golimumab	12	35.83	±17.57	3	37.31	±20.63	9	35.33	±17.80	0.695
	ixekizumab	14	143.35	±43.11	3	145.97	±64.65	11	142.64	±39.71	0.885
IL-4 (pg/ml)	Baseline	16	0.51	±1.24	3	1.70	±2.77	13	0.23	±0.44	0.030
	S. pyogenes	16	11.25	±3.00	3	12.99	±3.49	13	10.85	±2.88	0.250
	adalimumab	16	9.80	±2.83	3	11.61	±3.07	13	9.38	±2.72	0.282
	golimumab	12	9.77	±2.66	3	10.76	±2.82	9	9.44	±2.70	0.532
	ixekizumab	14	12.06	±2.90	3	13.26	±3.70	11	11.73	±2.76	0.434
IL-10 (pg/ml)	Baseline	16	9.14	±25.77	3	36.74	±59.53	13	2.77	±2.20	0.491
	S. pyogenes	16	809.13	±506.81	3	826.02	±295.05	13	805.23	±553.60	1.000
	adalimumab	16	397.43	±259.67	3	446.96	±242.58	13	386.00	±271.52	0.800
	golimumab	12	524.68	±186.08	3	421.73	±230.19	9	559.00	±170.49	0.282
	ixekizumab	14	943.91	±420.89	3	907.59	±364.74	11	953.82	±450.76	0.555
IL-13 (pg/ml)	Baseline	16	0.08	±0.25	3	0.07	±0.13	13	0.08	±0.28	0.350
	S. pyogenes	16	2.79	±1.75	3	4.21	±2.13	13	2.46	±1.56	0.339
	adalimumab	16	2.22	±1.33	3	2.18	±1.05	13	2.23	±1.42	0.954
	golimumab	12	2.59	±1.52	3	2.34	±1.26	9	2.67	±1.66	0.736
	ixekizumab	14	3.19	±1.70	3	4.23	±2.31	11	2.91	±1.51	0.418
IL-17 (pg/ml)	Baseline	16	3.29	±5.48	3	8.86	±11.75	13	2.00	±2.24	0.289
	S. pyogenes	16	92.77	±38.42	3	104.12	±46.72	13	90.15	±37.97	0.425
	adalimumab	16	89.34	±37.80	3	108.79	±47.11	13	84.85	±36.05	0.439
	golimumab	12	91.11	±39.92	3	97.11	±43.89	9	89.11	±41.13	0.864
	ixekizumab	14	92.03	±29.95	3	102.14	±46.58	11	89.27	±26.33	0.637

Mann-Whitney U test. **p* <0.05, ***p* <0.01.
