# Supplementary material for: Mechanisms of Tumor Necrosis Factor-Alpha Inhibitor-Induced Systemic Lupus Erythematosus
Source: Front Med (Lausanne). 2022 Jun 6;9:870724. doi: 10.3389/fmed.2022.870724 (PMC9208548; doi:10.3389/fmed.2022.870724)
Supplement: Supplementary file 2 [file Data_Sheet_2.pdf]

Appendix B

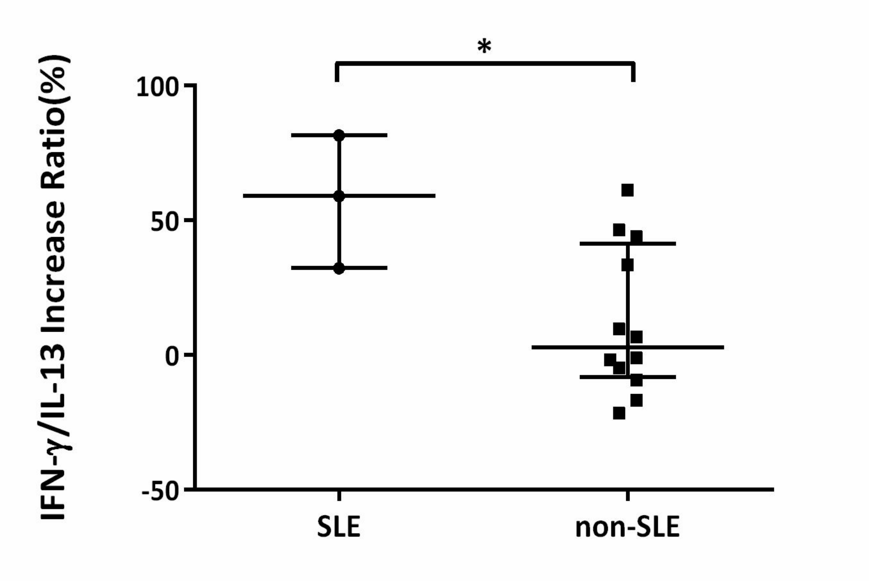

Fig. S1. Increased IFN-γ/IL-13 ratio in PSO and PSA patients with systemic lupus erythematosus (SLE). Three patients with SLE and four patients with PSO+PSA without SLE (P4, P6, P7, P8) and 15 treatment courses of biologics were as follow adalimumab, 6; etanercept, 1; golimumab, 1; ustekinumab, 3; secukinumab, 3; and guselkumab, 1. The ratio of IFN-γ/IL13 was significantly increased in the SLE group (*p* =0.02).
